# Evaluation of breastfeeding care and education given to mothers with low-birthweight babies by healthcare workers at a hospital in urban Tanzania: a qualitative study

**DOI:** 10.1186/s13006-020-00280-1

**Published:** 2020-05-06

**Authors:** Kyoko Tada, Yoko Shimpuku, Bruno Sunguya, Shigeko Horiuchi

**Affiliations:** 1 St. Luke’s International Hospital, 9-1 Akashi-cho, Chuo-ku, Tokyo, 104-8560 Japan; 2 Graduate School of Biomedical and Health Sciences, Hiroshima University, 1-2-3 Kasumi, Minamiku, Hiroshima, 734-8551 Japan; 3 Department of Community Health, Muhimbili University of Health and Allied Sciences, United Nation Road, Upanga, Ilala Municipality, Dar es Salaam, Tanzania; 4 St. Luke’s International University, 10-1 Akashi-cho, Chuo-ku, Tokyo, 104-0044 Japan

**Keywords:** Exclusive breastfeeding, Education, Resource-limited setting, Qualitative study

## Abstract

**Background:**

The total neonatal mortality in Tanzania remains high reaching as much as 44,900 deaths per year, particularly among low birthweight (LBW) babies. This makes Tanzania the fourth African country with the highest number of annual neonatal deaths. Studies have shown the advantages of breast milk for LBW babies and the effectiveness of interventions from healthcare workers (HCWs) to encourage mothers to achieve exclusive breastfeeding (EBF). Although these interventions can substantially reduce mortality in this vulnerable group, they remain insufficient in practice particularly in resource-limited countries. Therefore, there is an urgent need to establish the most appropriate interventions for mothers with LBW babies, particularly in these countries. To help address this need, we evaluated the breastfeeding care and education given to mothers with LBW babies by HCWs during hospitalization in Tanzania.

**Methods:**

A qualitative study using semi-structured interviews with mothers of LBW babies at an urban hospital in Tanzania was conducted. We assessed their understanding of breastfeeding at discharge. All the interviews were conducted in local Swahili and then translated to English. Data were analyzed using content analysis.

**Results:**

Among the 19 mothers interviewed, only four breastfed their baby within an hour after birth. Nine mothers received no support from HCWs when they breastfeed their baby for the first time. Ten mothers received no education on EBF, and there were mothers who misunderstood the EBF definition. Eight answered that they had difficulty breastfeeding their baby at discharge. Four mothers were dissatisfied with the care and education given by HCWs, and six mothers provided suggestions for improvements. Although six mothers had a high reliance on HCWs, they had difficulty asking HCWs questions because of their authoritative attitude and behavior.

**Conclusions:**

Mothers with LBW babies need special support to increase their ability to breastfeed and ensure EBF continuance. To address the gaps between the currently provided breastfeeding interventions and the ideal breastfeeding interventions, improvements in the quality and quantity of breastfeeding care and education are required. Training HCWs to systematize standard interventions, confirming mothers’ understanding, and ensuring a comfortable environment for mothers are absolutely needed.

## Background

Globally, the neonatal mortality rate has decreased from an estimated rate of 37 to 18 deaths per 1000 live births between 1990 and 2018 [1]. However, the number of annual neonatal deaths in Tanzania remains high reaching as much as 44,900, making Tanzania the fourth African country with the highest number of neonatal deaths [2]. This is particularly true among low birthweight (LBW) babies whose weight is under 2500 g at birth as defined by the World Health Organization (WHO). LBW babies compose a large population group at a rate of one out of 14 babies in the country [3, 4]. These babies are approximately 20 times more likely to die than heavier babies based on epidemiological observations [5]. This is considered to directly or indirectly contribute up to 80% of all neonatal deaths [6, 7].

Exclusive breastfeeding (EBF) substantially reduces the mortality of LBW babies [3, 8, 9]. Breast milk is known to have advantages for LBW babies. Moreover, healthcare workers’ (HCWs’) interventions have been shown to effectively encourage mothers to achieve exclusive breastfeeding. In developing countries, however, HCWs’ interventions remain insufficient. Mothers face problems breastfeeding their babies at home because of their insufficient guidance during hospitalization [10, 11], particularly when their babies are premature with physiological limitations to breastfeed [12]. Delayed initiation of breastfeeding and a long period of mother-child separation after birth may also affect the milk production of mothers of LBW babies [13]. This may decrease their confidence because of the perception of insufficient breast milk production. Encouraging mothers to express breastmilk early after birth is another important support to increase the breastmilk production of mothers whose babies are unable to breastfeed effectively, especially during the first hour after birth [14–16]. In Tanzania, the median duration of EBF for all babies is only 3 months, and this rapidly decreases with the age of the baby [4], contrary to the recommendations and the global target of 50% of babies at 6 months of age [17]. Only 27% of all babies aged four to 5 months are exclusively breastfed compared with 59% of all babies aged 2–3 months and 84% of all babies aged 0–1 month. For breastfeeding practice by background characteristics, the National Bureau of Statistics found that mothers in rural areas are more likely to exclusively breastfeed than those in urban areas [4].

Therefore, appropriate care especially during the first critical days of life of high-risk babies is urgently needed. Although there have been studies on interventions in mothers with LBW babies regarding infant feeding [6, 18], there is no sufficient evidence that contextualizes the reality of interventions from HCWs in settings that can improve postnatal breastfeeding care.

We conducted this study to evaluate the current breastfeeding care and education interventions being provided by HCWs to mothers with LBW babies in a resource-limited setting at a hospital in urban Tanzania. A comparison of these current interventions with the recommended interventions may contribute to the establishment of the most appropriate method of educating Tanzanian mothers with LBW babies to reach their target EBF rate and prevent neonatal deaths.

## Methods

### Aims

To evaluate the breastfeeding care and education interventions provided by HCWs to Tanzanian mothers with LBW babies during hospitalization, and to identify areas for improvement to enhance the EBF rate.

### Study design

This study used a qualitative study design involving semi-structured interviews with mothers of LBW babies regarding the breastfeeding care and education interventions that they received from HCWs during their hospitalization, as well as their breastfeeding knowledge at discharge.

### Study setting

The study was conducted at a metropolitan hospital in Dar es Salaam, Tanzania. Two units were chosen as LBW babies in the hospital are admitted either in the neonatal unit or kangaroo unit. *The neonatal unit* has 130 baby cots and admits between 12 and 25 neonates per day up to 28 days of life. Admitted babies include (a) LBW babies (under 2500 g at birth) such as premature and small for gestational age babies who may be normal or sick, (b) normal term babies whose mothers are sick or not able to care for their babies, and (c) babies who need treatments. The neonatal unit has two shifts with five to six nurses on duty during a day shift, and two to four nurses on duty during a night shift. The shortage of nurses was severe, as dead babies were often just covered with white bed sheets laid for a long time in between living babies. Babies in the neonatal unit rarely have options for kangaroo mother care or skin-to-skin contact with their mothers.

*The kangaroo unit* admits mothers and stable babies weighing < 1500 g to provide kangaroo mother care. The kangaroo unit has 30 beds and admits between 20 and 40 neonates per month. The number of admission days is within 2–40 days wherein three nurses are on duty in the day-time shift. To provide kangaroo mother care as a substitute for incubators, mothers lay their baby on their chest for almost 24 h except when they eat, feed their baby, or take a bath.

### Sampling

Patton suggested that purposeful samples can be stratified or nested by selecting particular units or cases that vary according to a key dimension [19]. Moreover, triangulation of data sources, that is, to examine the consistency of different data sources from within the same method, is considered to enhance trustworthiness in qualitative research [19]. Therefore, a purposeful sampling strategy with triangulation of data sources was used for recruitment by inviting mothers with LBW babies from two different wards. Eleven mothers were from the neonatal unit and eight mothers were from the kangaroo unit, with the key dimension of breastfeeding care.

The inclusion criteria of potential participants were mothers with LBW babies who were about to leave the ward, who could breastfeed (i.e., nurse their baby directly from their breasts) or feed their baby using alternative ways (e.g., cup feeding), who could talk and read Swahili language, and who lived in Dar es Salaam. Mothers of babies with underlying disease (e.g., HIV, birth asphyxia, disability, congenital anomalies, and infections), mothers with babies weighing less than 1000 g, or mothers who were unable to breastfeed at discharge were excluded.

The researcher (KT) defined EBF according to the WHO definition in the following context: an infant receives only breast milk (including expressed breast milk or breast milk from a wet nurse) and is allowed to receive only oral rehydration solution, drops, and syrups (i.e., vitamins, minerals, and medicines) [20].

#### Recruitment

The researcher informed the nurses in charge of the two units regarding the inclusion and exclusion criteria of this study. The researcher then requested the nurses to go through all the patients’ files placed in the nursing station to identify all the mothers who meet the criteria as study participants. The target participants were then informed directly by the researcher and a translator about the study’s purpose, methods, and ethical considerations that cover re-examinations after being discharged to home. A participation request form with a consent/refusal form was provided. When a mother consented to participate, she signed the consent form. The mothers were informed that their non-participation or declining after their initial participation would not influence any of their future care at the facility after being discharged such as in their follow-up examinations. The nurses were not informed whether the participants consented to participate in the interviews or refused.

#### Data collection

Data were collected from April 2017 to June 2017 by semi-structured interviews of the mothers in the Swahili language. The researcher (KT) who is a non-native Swahili language speaker was the main interviewer, and the research assistants who were native Tanzanians about to complete their nursing degree provided support when the researcher faced language difficulties. Data were collected until saturation, the point at which no new relevant information is forthcoming even if more people are interviewed [21]. The mothers were interviewed in a private room outside the ward and could not be seen by the nurses. The interviews lasted from 30 to 60 min and an interview topic guide was used. The interview guide was developed by the researcher on the basis of previous reports [22–24] and met the study purpose. The interview guide asked about (a) the sociodemographic characteristics of the mothers, as well as the breastfeeding care and education they received from HCWs, (b) whether they started breastfeeding within an hour after birth, and (c) their knowledge of breastfeeding. The interview guide was translated into the Swahili language which is familiar to most Tanzanians.

#### Ethical consideration

The study was conducted based on the principles of ethics such as harmlessness, voluntarily participation, anonymity, and protection of privacy and personal information. The interviews were conducted in a private room outside the ward. The participants were informed that all information they will provide would be used for this study only.

All the interview data were recorded upon the consent of the participants using a voice recorder. The recorded data were transcribed as electronic data using a personal computer and were kept and managed using a password. The voice recorder, field notes, and computer used in this study were kept in a private locker for security purposes. Identifiable data such as names were replaced with ID numbers and kept in a separate place. Anonymity was strictly maintained and the participants were never identified. The research assistants, who supported the interviews and transcribed the data, were informed about the study and confidentiality by the researcher. Confidentiality was obliged from the research assistants by signing a confidentiality agreement.

### Analysis

Each interview was recorded, transcribed, and translated from Swahili to English using Microsoft Word before analysis. The transcriptions were carried out by the researcher and research assistants. The demographic data of the mothers were summarized using a number and percentage. Content analysis was used to analyze the data [25] by highlighting words, phrases, and sentences that described (a) the care and education the mothers received from HCWs, (b) mothers’ perceptions of the breastfeeding education, and (c) mothers’ knowledge of breastfeeding. Data were then coded and grouped into categories. Data analysis was overseen by faculty supervisors.

## Results

### Sociodemographic characteristics of study participants

A total of 19 mothers were interviewed just before their discharge. Eleven mothers were from the neonatal unit and eight mothers were from the kangaroo unit. Data were collected until saturation. The age of the mothers ranged from 16 to 38 years (average, 25 years). Nine mothers had only primary school education, eight mothers had secondary school education, one mother graduated from college, and one mother had no education. Thirteen mothers were married and six mothers were not. The mothers had an average of 1.8 births until the interview, ranging from one to four total births.

Regarding family structure, the number of people living together ranged from three to six (average, 4.3). Ten participants lived in nuclear families and other participants lived with their relatives, of which only two families were with their mother-in-law. Eleven mothers were Christians, and eight were Muslims. Thirteen mothers were working before giving birth and six mothers were housewives. Among the mothers who were working, five mothers were on their maternity leave and the other mothers resigned from their jobs.

Ten mothers planned their pregnancy, and nine mothers did not. The gestational age ranged from 28 to 36 weeks (average, 32 weeks), which were all classified as preterm [26]. Fifteen mothers had spontaneous vaginal delivery (SVD) and four mothers had cesarean section (CS). Fourteen mothers gave birth at the metropolitan hospital, three at regional referral hospitals, one at a clinic, and one at home. All mothers, except for one who gave birth at home, had assistance by HCWs.

### Breastfeeding care and education received by mothers from HCWs

#### Care related to early breastfeeding soon after birth

Of the 19 mothers interviewed, only four mothers were able to breastfed their baby within an hour after birth. The reasons why the other mothers were not able to initiate early breastfeeding were categorized as follows: “baby’s physical problems,” “mother-infant separation,” and “misconception.” The primary reason was the baby’s physical problems, such as the need for oxygen therapy, having a nasogastric tube or drip, and immaturity to suck.

“*After delivery, because my baby didn’t breathe well, he was quickly taken down stairs [another ward] to be given oxygen. I delivered on Monday, so Tuesday, Wednesday, Thursday had passed. I started breastfeeding on Friday, when he was 5 days old. (Mother N-1).*”

The second common category was mother-infant separation, which included both mothers who had SVD and mothers who had CS. All mothers who had CS failed to initiate early breastfeeding.

“*I was not given the baby the day I gave birth because I delivered by operation, after that I was brought here and my baby was taken to ward #; the second day I started breastfeeding. (Mother N-7).*”

Aside from these reasons, a mother who had misconception stated the following:

“*I did not breastfeed] because the baby didn’t poo black stool. When the baby is born he/she must have black stool. After when the baby has black stool is when you can give food . . . yeah they [nurses] told me . . . this baby didn’t eat for two days. (Mother K-7).*”

#### Education related to breastfeeding

The quality and quantity of education the mothers received depended on the HCW’s availability and individual skills, which were not standardized. They received education either in a group or in person. The mothers who received personal education described their educational experience as more of a coincidence than a planned education as follows:

“*The education itself is not that someone (nurse) is coming to teach us, but it is like chatting only. (Mother N-3).*”

Comparing the two units, the mothers admitted to the neonatal unit showed a higher percentage of insufficient education (Table 1). The percentage of mothers who did not receive all three items about breastfeeding, namely, the definition of EBF, how to express breast milk, and how to cup feed, was 90.9% (10 out of 11 mothers) in the neonatal unit and 75% (six out of eight mothers) in the kangaroo unit.

**Table 1:** Care and education received by mothers and their understanding

Unit	Care		Contents of education			Knowledge of mothers		
	Feeding method^**a**^	First time support^**b**^	EBF (definition, duration)	Expressing breast milk	Cup feeding	EBF		Breast stimulation and milk production^**c**^
						6 months	No water	
N-1	BF	○	○	○	○	○	×	×
N-2	Cup	×	○	×	○	○	○	○
N-3	BF	○	○	×	×	○	○	○
N-4	NG	○	○	×	×	○	○	N/A
N-5	BF	×	○	×	×	○	○	○
N-6	BF	○	×	×	○	○	○	×
N-7	BF	○	×	×	×	×	×	○
N-8	BF	○	×	×	×	○	×	×
N-9	BF	×	×	×	×	○	×	○
N-10	BF	×	×	×	×	○	×	×
N-11	BF	×	×	×	×	○	○	×
K-1	BF	○	○	○	○	○	○	×
K-2	BF	○	○	○	○	×	×	×
K-3	NG	○	○	×	○	○	○	×
K-4	Cup	○	×	○	○	○	○	×
K-5	BF	×	N/A	○	×	○	×	○
K-6	Cup	×	×	×	○	○	○	×
K-7	BF	×	×	×	×	○	○	○
K-8	Cup	×	×	×	×	×	○	○

^a^ Procedure used at the first time of breastfeeding

^b^ Support the mothers received by HCWs at the first time of breastfeeding

^c^ Association between frequent breastfeeding and milk production

*N/A* data not available, *BF* breastfeeding, *Cup* cup feeding, *NG* nasogastric tube feeding, *EBF* exclusive breastfeeding, × not received, did not know; ○ received, knew

#### Support the mothers received from HCWs at their first time of breastfeeding

Nearly half the mothers did not receive any care at their first time of breastfeeding (Table 1). The mothers who had no support fed their baby either directly from their breasts or directly from a cup by “looking at pictorial instructions on walls”, “watching how others do”, or “from their own experiences of feeding milk to their older children”.

“*I stayed for half an hour at the labor ward, then I was given my baby, then I started breastfeeding my baby. I had no support. They didn’t tell me [how to breastfeed], I watched how other mothers breastfeed, then I started breastfeeding. I felt good but the baby didn’t breastfeed well. (Mother N-11).*”

Two mothers, who also had no support, finally received advice from HCWs when they found the mothers positioning their baby inappropriately.

“*A nurse showed me, because I was holding the baby like this inappropriate latch on position, then the nurse told me and showed me [how] to hold my baby in a right way. (Mother N-3).*”

Other mothers had support from the beginning on how to breastfeed their baby.

*“The doctor directed me how to handle the baby and to breastfeed. I felt good. (Mother K-2).”*

#### Education on EBF

Ten mothers did not receive education on EBF.

“*To be honest, I was not taught by anyone because I gave birth on 27*^*th*^*, then I was discharged on 28*^*th*^*, so I was not given any advice, but I was asking other breastfeeding mothers how they breastfeed their babies. (Mother N-11).* ”

Of the mothers who received education on EBF, there were differences in how they understood its meaning. For example, when the mothers were asked to describe in their own words what EBF means, there were mothers who could not answer correctly either the months of the recommended EBF period or if water is allowed during the EBF period (Table 1).

“*They said EBF is very good for the baby. [For how long?] I don’t know, maybe 8 months? (Mother K-2).*”

On the other hand, the mothers who understood EBF well answered,

“*Yes, they told us. They [nurses] told us not to give them [babies] anything such as water and food other than breast milk until they are six months. (Mother N-2).*”

#### Education on expressing breast milk and cup feeding

Fourteen mothers did not receive education on expressing breast milk, of whom ten were the mothers admitted to the neonatal unit. Eleven mothers did not receive any explanation about cup feeding. Among the mothers of the six babies who were unable to breastfeed directly, only one mother was taught how to express breast milk (Table 1).

#### Education on ways to increase breast milk production

Regarding ways of increasing milk production, the mothers were instructed to drink hot tea, eat nutritious foods, or compress the breast with hot water.

“*They told us if you don’t have milk, eat foods which will increase milk, like grand nuts, porridge, and ginger tea. (Mother K-2).*”

“*We were told that when we go home and if the milk would not come out, we should take hot water and compress my breast with that hot water until milk comes out. (Mother N-10).*”

#### Knowledge (misconception) of the association between frequent breastfeeding and milk production

Only eight mothers knew the association between frequent breastfeeding/expressing breast milk and milk production (Table 1).

“*We were given a seminar that told us that when you deliver a baby try to breastfeed frequently, as production of milk will increase by stimulating your breast. (Mother N-3).*”

“*They say when the baby start breastfeeding the ducts opens and milk start to come out. (Mother N-7).*”

Eleven mothers had either misconception or did not know such association (Table 1).

“*If you express many times milk will block. (Mother K-2).*”

### How mothers felt about breastfeeding and the given education

#### Difficulty in breastfeeding their baby at discharge

Eight out of the 19 mothers answered that they had difficulty latching on the baby to their breasts.

“*She is weak in sucking when I see lots of milk coming out, she chokes, so I remove her from the breast. (Mother N-10).*”

“*To put the nipples on her mouth, she struggles. (Mother N-1).*”

#### Authoritative attitude and behavior of HCWs

Six mothers answered that they had difficulty in asking HCWs questions while hospitalized because of their authoritative attitude and behavior.

“*Sometimes they get tired when you ask them, they say that it is not their job, you can find a woman crying. Other mothers do it in their own way because whenever they ask nurses, they are told “It’s not my job”. (Mother N-9).*”

"*I do ask, but sometimes I feel coward because when you ask then they abuse, they harshly respond. I have tried to ask once, when the baby needed the tube for feeding. I asked her that “my baby can’t swallow, the tube is out, can you come and put it again? I want to give him milk.” She replied, “Do you think inserting tube to your nose is not painful? Let me insert to yours so that you can feel how much it is painful. (Mother N-2).*”

### Satisfaction, dissatisfactions, and suggestions about the care and education given by HCWs

Of the 19 mothers interviewed, four mothers did not receive education from HCWs. They were not satisfied with this experience and gave suggestions to improve the education given by HCWs. They suggested that HCWs 1) “provide information about the risk of not achieving EBF,” 2) “confirm understanding of individual mothers,” 3) “allocate time for education or identify a HCW responsible for education,” 4) “emphasize things that are important,” and 5) “educate family members.”

1. Suggestion regarding the risk of not achieving EBF:

“*My suggestion is that when we give birth, they have to advise the mother what she should do about caring for the baby, something should be done like this … or the effects of not breastfeeding exclusively, or not doing something to the baby which is very important to the child, that is what is my advice. (Mother N-8).*”

2) Suggestion on confirming the understanding of individual mothers:

“*I think they should not get tired because we are different people, others might know but others might not know, so when we didn’t understand something and ask them, they should not get tired. (Mother N-9).*”

“*They should make mother’s understanding better . . . they needed to put more of their effort [in teaching], because everyone has his/her own understanding. (Mother N-6).*”

3) Suggestion with regard to HCWs allocating time for education or identifying a HCW responsible for education:

“*My suggestions is that they have to plan a certain time [for education], for example the time when mothers go to breastfeed their baby [to the ward], they should explain in detail about breastfeeding, but if they [nurses] have a lot of things to do, there must be one person from any unit who can advise mothers, a person who can come and teach mothers how their breast milk help our baby, or to advise us how we are supposed to care our baby who are underweight so that they will become okay. (Mother N-1).*”

4) Suggestion on emphasizing things that are important:

“*What I advise is that when the mothers ask questions, don’t hide the answers and tell them all what you know, then the doctor or the nurse should tell them and emphasize them in order to make them aware, this is because some mothers when they ask, they are still not aware. (Mother N-5).*”

5) Suggestion on educating the family members:

*“When I go back home, people may complain that I am not working . . . and just staying in the room. Therefore, one relative should also be educated that mothers should stay [in the kangaroo position] with her baby all the time . . . because when I go back home, they can start saying that I just had normal delivery and is staying in the room all the time [doing nothing]. That’s why my mother should come and be educated in order to understand [my situation], so that even if I go home, they know. (Mother K-3).*”

## Discussion

The importance of breastfeeding intervention after birth in improving EBF behavior has been suggested [27]. Such intervention may consequently reduce the neonatal mortality rate [3, 8, 9, 27–30]. However, in reality, even leading national hospitals in Tanzania face difficulty in introducing the recommended interventions, so that EBF remains at a low level. The present results expressed the real voices of Tanzanian mothers regarding what HCWs should consider in terms of breastfeeding care and education to better meet the needs of Tanzanian mothers and possibly provide ideal recommendations for similar resource-limited settings worldwide.

### Gaps between ideal care and provided care: failure of early initiation of breastfeeding soon after birth, delaying expression of breast milk, and insufficient care at the first time of breastfeeding

The WHO advocates that babies capable of breastfeeding should be placed in the mother’s breast as soon as they are clinically stable after birth [3]. This promotes the early initiation of breastfeeding after birth, which is significantly associated with increased milk production and continuous EBF behavior [14]. For mothers whose babies are unable to suck effectively, expressing breast milk is recommended preferably within six hours after birth for continuous exclusive breastfeeding [31, 32]. Moreover, the early initiation of breast milk expression following birth has been shown to increase milk production in mothers with LBW babies. Initiation within an hour was significantly associated with milk volume after 1 week of birth [14]. An extended duration from birth to expressing breast milk was negatively associated with milk volume [33]. Mothers who frequently expressed their breast milk, as much as seven times or more per day, produced a significantly high average volume of milk per day [34].

Of the 19 mothers interviewed, 15 mothers failed to initiate breastfeeding early. Although the early initiation of breastfeeding may depend on the baby’s condition, situations such as mother-infant separation should be avoided. A long period of mother-baby separation can have negative effects on the milk production of mothers [13]. This may accelerate the use of alternative milk products or fluids for their babies.

Incidentally, only about half of the mothers received care at the first time of breastfeeding their baby. The other mothers were not provided education or were only given education when the HCWs found them by chance breastfeeding in an inappropriate way. Eight mothers mentioned that they lack the confidence to breastfeed at discharge, implying that the absence of interventions may contribute to the decline in the confidence of mothers to breastfeed. Brockway et al. [35] reported that improving breastfeeding self-efficacy is one of the key factors for achieving continuous exclusive breastfeeding. Thus, HCWs need to intervene in ways that will enhance the confidence of mothers to breastfeed. The use of a protocol that includes measures for avoiding mother-baby separation and for promoting early breastfeeding with care should also be reconsidered.

### A key to educating mothers is not only to provide education but also to enhance their understanding

To ensure the continuance of EBF, mothers should have both EBF knowledge and confidence in breastfeeding [30]. In addition, they should know how to express breast milk and cup feed, particularly those with LBW babies who have low ability to breastfeed. Moreover, it is important for mothers to understand that frequent breastfeeding or expression of breast milk helps to increase breast milk production. This is because mothers with LBW babies occasionally have less breast stimulation which results in low milk production [33]. Unfortunately, only one mother out of six who needed to express breast milk was taught how to by HCWs. More than half of the mothers did not understand the association of frequent breast stimulation with milk production.

Based on the interviews, the mothers received insufficient education before discharge. The majority of the mothers missed receiving information on EBF, expressing breast milk, cup feeding, and the association between frequent breastfeeding/expression of breast milk and milk production.

An important suggestion by the mothers is for HCWs to carefully assess the understanding of each mother during the intervention, as some mothers have indicated that they did not understand correctly despite receiving education from HCWs. Therefore, it is crucial for HCWs to know that what is important in education is not only the act of intervention but also the sufficiency of understanding of the mothers.

Six mothers experienced difficulty in asking questions to HCWs because of their authoritative attitude and behavior. If this situation remains unchanged, mothers will lose their chance of learning about breastfeeding before discharge. As previously reported, relaxation is associated with a high volume of expressed milk [36, 37]. Thus, providing a comfortable environment to mothers during hospitalization is imperative.

A systematized education with alternative options is also recommended to prevent omissions of contents. This is because the contents of breastfeeding education are extensive and difficult to effectively teach. Although previous studies have indicated that birth in health facilities show a positive association with EBF practices [38–40], the present findings indicate that birth in a health facility alone is insufficient in enhancing exclusive breastfeeding. This means that the effectiveness of education in facilities depends on whether it meets the mothers’ individual demands. A specific example is for mothers who had CS. They are likely to suffer from wound-induced pain. Thus, they need to be educated that the preferable position while breastfeeding is to hold their baby in a position that avoids the wound. This guidance provides mothers knowledge of alternative positions aside from the cradle position alone wherein they hold their baby over the wound.

### Strategies for improving the quality of breastfeeding care and education for mothers with LBW babies

An important strategy for improving the quality of breastfeeding care and education for mothers with LBW babies is to standardize breastfeeding care and education. This can be achieved by making a checklist to follow to prevent content omissions, and then taking the improvements into consideration for mothers with LBW babies. Each of the contents should be described in detail with alternatives.

It is conceivable that the shortage of nurses in most healthcare facilities in Tanzania would result in the lack of care that can be given by nurses. This consequently implies the limited capacity of nurses to provide education. Thus, it is recommended that effective group educational materials be made so that no mothers will be left out. The contents should include the following: (a) early initiation of EBF with instructions on how to breastfeed the baby at the first time, including positioning and latch-on, (b) emphasis on two key points for EBF: “other fluids and water or honey are not allowed” during the first “6 months” period, (c) how to express breast milk, (d) how to cup feed, including the reasons for the need to feed by a cup, (e) the relationship between stimulating the breast by breastfeeding or expressing breast milk and milk production, as well as how to increase milk production, and (d) the risks of not achieving EBF and the benefits of EBF for the baby and mother.

Regarding the HCWs, despite the difference of education revealed between the neonatal unit and the kangaroo unit, the quality and quantity of care and education provided to the mothers were not standardized. They should have up-to-date knowledge, be trained to use new techniques, and be given full support to increase their confidence on how to adequately educate mothers about EBF and breastfeeding. All HCWs should have a commitment to play an important role in encouraging mothers who breastfeed. Making a comfortable environment is also necessary so that mothers can easily ask HCWs about their troubles without fear. Effective communication may also improve mothers’ satisfaction, as emphasized by WHO during postpartum care. In terms of confidence, Kwah et al. stated that confidence in HCWs improves the quality of interventions in practice [41].

Taken together, a highly effective and systematized approach to postpartum care must be carefully implemented. This approach should include the following elements: re-training of HCWs on how to support mothers to breastfeed, assessment of the need of giving expressed milk in addition to breastfeeding according to the baby’s weight and ability to suck, and how and what to observe when shifting from cup feeding to breastfeeding regardless of the baby’s age.

### Strengths and limitations of this study

Utmost effort was observed in maintaining scientific rigor throughout the study based on the framework, credibility, dependability, and transferability criteria espoused by Lincoln and Guba [42]. Credibility was ensured throughout the process by the careful guidance of the faculty supervisors (YS, SH) who have extensive experience in research and publications in this field. The faculty supervisors provided substantial contributions to the conception and design of the study, analysis and interpretation of data, critical revisions of the manuscript for important intellectual content, review of the final version of the manuscript, and careful checking of the accuracy and integrity of all parts of the present work. Dependability and confirmability were demonstrated by the provision of an external audit as overseen by the faculty supervisors (YS, SH). External validity and transferability were verified by a faculty supervisor (BS). The findings included sufficient “thick description” for readers to assess the potential transferability and appropriateness for their own setting.

The limitations of this study were as follows. *First*, the evaluation was limited to only a single hospital in an urban area in Tanzania. Although the present results reflected the trend of LBW babies with mothers who can potentially provide care for their babies and with no underlying disease in the urban district of Dar es Salaam, the actual condition of the babies show a highly vulnerable population, and likely remains much more uncertain in rural districts.

*Second*, social desirability bias cannot be completely ruled out. Although the researcher avoided revealing that she was a nurse during the interview, the participants may have over-reported their EBF behavior to please the researcher or to try to sound good, which reflects the element of social desirability bias.

The strengths of this study were as follows. *First*, the prospective approach was considered to increase the accuracy of the mothers’ answers to the interview compared with the accuracy of the mother’s answers based on recall. *Second*, the researcher had volunteered for more than 1 year in Tanzania before conducting this study; thus, prolonged engagement and persistent observation as suggested by Lincoln and Guba have already been initiated [42]. *Third*, this is one of the few studies in Tanzania that provides detailed data about mothers’ perspectives regarding the level of breastfeeding support and education that they receive from HCWs and their suggestions for improvements.

## Conclusion

Mothers with LBW babies need special attention and support in terms of their ability to breastfeed to ensure achievement of the recommended EBF rate and to help reduce the neonatal mortality rate. This study identified the gaps between the currently provided breastfeeding care and education interventions and the ideal breastfeeding care and education interventions recommended by WHO and local Tanzanian mothers with LBW babies in a resource-limited setting. To address these gaps, improvements of breastfeeding care and education are required in terms of both quality and quantity, focusing on the mentioned key factors for achieving continuous EBF. In addition, it is necessary to train HCWs to systematize the standard care and education interventions, confirm each mother’s understanding, and ensure a comfortable environment for mothers.

## Data Availability

The dataset or transcripts are available from the corresponding author upon reasonable request.

## Abbreviations

CS: Cesarean section
EBF: Exclusive breastfeeding
HCW: Healthcare worker
LBW: Low birth weight
SVD: Spontaneous vaginal delivery
WHO: World Health Organization
